# Evaluation of the Resolution in Inverse Scattering of Dielectric Cylinders for Medical Applications

**DOI:** 10.3390/s23167250

**Published:** 2023-08-18

**Authors:** Ehsan Akbari Sekehravani, Giovanni Leone

**Affiliations:** Department of Engineering, University of Campania “Luigi Vanvitelli”, I-81031 Aversa, Italy; giovanni.leone@unicampania.it

**Keywords:** linear inverse scattering, number of degrees of freedom, point spread function, inhomogeneous medium, resolution, TSVD inversion, localization, breast cancer imaging

## Abstract

The inverse scattering problem has numerous significant applications, including in geophysical explorations, medical imaging, and radar imaging. To achieve better performance of the imaging system, theoretical knowledge of the resolution of the algorithm is required for most of these applications. However, analytical investigations about the resolution presently feel inadequate. In order to estimate the achievable resolution, we address the point spread function (PSF) evaluation of the scattered field for a single frequency and the multi-view case both for the near and the far fields and the scalar case when the angular domain of the incident field and observation ranges is a round angle. Instead of the common free space condition, an inhomogeneous background medium, consisting of a homogeneous dielectric cylinder with a circular cross-section in free space, is assumed. In addition, since the exact evaluation of the PSF can only be accomplished numerically, an analytical approximation of the resolution is also considered. For the sake of its comparison, the truncated singular value decomposition (TSVD) algorithm can be used to implement the exact PSF. We show how the behavior of the singular values and the resolution change by varying the permittivity of the background medium. The usefulness of the theoretical discussion is demonstrated in localizing point-like scatterers within a dielectric cylinder, so mimicking a scenario that may occur in breast cancer imaging. Numerical results are provided to validate the analytical investigations.

## 1. Introduction

The inverse scattering problem requires determining the physical and geometric characteristics of an unknown object from scattered field data provided via the induced perturbation of known incident fields. Since the inverse scattering problem is nonlinear, approximations such as the Born [1] or Rytov [1,2] ones for dielectric objects and physical optics (PO) [3,4] approximation for metallic objects can provide a linear relationship between the scattered field data and the scattering object.

The imaging method based on inverse scattering has gained significant interest and has been thoroughly researched. This is because of its flexibility and appropriateness for various applications, such as radar imaging [5], through-the-wall imaging [6,7], ground penetration radar (GPR) applications [8,9], biological imaging applications [10,11], breast cancer imaging [12,13,14,15], brain stroke detection [16,17], and medical imaging [18]. Far and near field data can be available according to the application; in particular, medical imaging can be accomplished in the near zone, which may provide better-resolution benefits compared with far-field imaging.

The full view is a typical case in inverse scattering, which arises as the incident fields illuminate the object from all angles, and the scattered fields are observed at all angles. This case is suitable for various industrial applications or medical imaging applications like head and breast imaging.

The achievable resolution refers to the ability to accurately distinguish and locate small features or details within an object or scene being imaged. It is related to the smallest resolvable distance or size of the features that can be distinguished with the imaging system and has raised much interest in microwave imaging.

In [19], based on nonlinear modelling and inversion, a super resolution was demonstrated for a near-field experimental microwave tomography system; however, in nonlinear inverse scattering, the results depend on the scattering scenario and cannot be investigated a priori. In [20], the resolution within a linear scattering model was considered; however, since the internal field is numerically computed as the exact one instead of being approximated by the incident one as in the Born approximation, no analytical discussion can be performed and it should be computed numerically. In [21], an alternative definition of resolution was introduced, but a numerical computation is always required for a comparison of different scenarios. An approach based on deep learning has been proposed in [22] to improve the spatial resolution for microwave imaging. Therefore, all these approaches achieve results about resolution based on numerical computations.

Instead, in linear scattering, the achievable resolution can be defined in terms of the point spread function (PSF) of the system and it represents the reconstruction of a point-like object. For a compact linear operator, singular value decomposition (SVD) can be used to introduce the PSF. The concept of PSF has found extensive usage in [23,24,25,26,27], showing its broad applicability and relevance in various fields.

The analytical evaluation of the exact PSF can be performed for a limited number of scattering geometries. For most scenarios, numerical methods are the only option. The truncated SVD (TSVD) [28] algorithm can be used to obtain the exact PSF. To compute the exact main lobe width of the PSF, an appropriate truncation value must be chosen, as using an incorrect value may affect the main lobe width and the side lobes. An appropriate truncation choice could be the number of degrees of freedom (NDF) of the scattering object, which is defined as the number of significant singular values of the pertinent scattering operator as they usually decay exponentially.

The NDF evaluation of the scattered field has been addressed in [29] for simple strip geometries for the full-view case in the far zone. That study has demonstrated that the same NDF can be obtained through the use of different variables. One way to provide an analytical expression of the PSF is by evaluating an approximation of the exact PSF to eliminate the limitations of the exact PSF. For instance, in [30], the evaluation of the approximated PSF has been considered for strip source/scatterer geometries for the full view. Those authors used the NDF as a truncation value in the TSVD algorithm. The results showed that the resolution is constant for the full-view case, the approximation worked well in the main lobe, and the NDF was a good choice for truncation. The same analysis is available in [31] for circumference source/scatterer geometries, and the analytical estimation of the NDF was also evaluated.

The NDF of the radiated field was considered in [32] for square sources, and the authors showed that the NDF of a full 2D square is equal to the NDF of a void square source. Sometimes, the analytical estimation of the NDF cannot be evaluated and it should be computed numerically. For the aspect-limited case, the NDF of the scattered field was computed numerically in [33] for curve geometries in different modalities. The analytical approximation of the PSF was also evaluated to estimate the resolution, and it was shown that the resolution is not constant.

A theoretical study on the achievable resolution and image quality of microwave imaging systems has been addressed in [34]. That study clarifies the relationship between resolution and limited-view versus full-view antenna array geometry, monostatic versus multistatic configuration, single-frequency versus wideband operation, and near-field versus far-field imaging. The theoretical relations of image resolution have been addressed in [35] for both the full-view and aspect-limited cases.

Most of the scattering scenarios assume the free space as being in the background, i.e., a homogeneous medium. In subsurface imaging for GPR applications, a two-layered medium has been taken into consideration [36,37,38] because the closed-form analytical expression of the scattered field can be found. In this circumstance, the available data are inherently aspect-limited to a half-space. Less attention has been paid to other layered geometries, although the closed-form expression of the pertinent scattered field can be available. In this paper, we are interested in a two-layered cylindrical medium composed of a homogeneous dielectric cylinder with a circular cross-section, embedded in the free space. This scenario can provide a theoretical reference for those applications, such as breast cancer/tumor imaging when scattering objects are located within a dielectric medium of known parameters. In this paper, we address the evaluation of the PSF of the scattered field for the single-frequency case and the multi-view sensing configuration for the full-view case to estimate the achievable resolution for both the far and near fields. We investigate how the permittivity of the dielectric can affect the truncation level and the resolution. In addition, an analytical approximate of the exact PSF is evaluated. Furthermore, a localization application that can be used in breast cancer/tumor imaging is provided. Numerical comparisons for the truncation index and two PSFs are provided to validate the theoretical discussion.

The plan for this paper is as follows: In Section 2, the problem statement, a PSF evaluation, and a discussion about how to choose the truncation level for a general scattering geometry are presented. Section 3 introduces and investigates the approximated PSF. Section 4 provides some numerical examples to validate the theoretical discussions. In Section 5, a numerical application to a localization problem is shown. Finally, in Section 6, conclusions are provided.

## 2. Statement of the Problem

**Figure 1:**
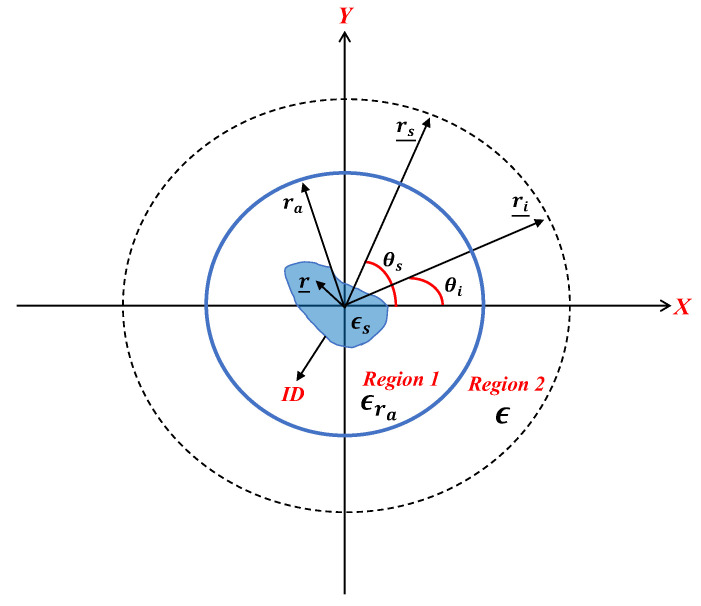
A pictorial view of the geometry of the problem. The dotted line indicates the positions of the source and receivers.

The general geometry of the problem is depicted in Figure 1. An unknown scatterer with relative permittivity $\varepsilon_{s}(\underline{r})$ is located within a domain referred to as the investigation domain (ID), which is embedded in a homogeneous dielectric cylinder (region 1) with a circular cross-section, radius $r_{a}$, and relative permittivity $\epsilon_{r_{a}}$, centered at the origin. The dielectric cylinder is located in a free space (region 2) with permittivity ϵ  = $\epsilon_{0}$ and both regions are nondispersive, while the magnetic permeability everywhere is equal to $\mu_{0}$. (The external medium can be also assumed to be different, though always homogeneous, provided that the appropriate dielectric permittivity is accounted for within the electromagnetic scattering model.) Accordingly, the background medium is inhomogeneous, as it consists of a cylindrically stratified medium.

Let us define as $\underline{r}=(\rho ,\phi )$, $\underline{r_{i}}=(r_{i},\theta_{i})$, and $\underline{r_{s}}=(r_{s},\theta_{s})$ as the position vectors spanning the scattering object, the source point (transmitter), and observation point (receiver), respectively. Hereafter, $r_{i}$ and $r_{s}$ are also assumed to be constant, i.e., they are circumference. In this paper, we consider the full-view case where the angular ranges of the excitation and observation angles are 2π wide, i.e., $-\pi <\theta_{i},\theta_{s}<\pi$. 

Under the Born approximation, in the scalar case, the only component of the scattered field is defined by

(1) $$E_{s}\left(\underline{r_{s}},\underline{r_{i}}\right)=\iint_{ID}^{}\chi (\underline{r})\;G_{s}\left(\underline{r},\underline{r_{s}}\right)E_{i}\left(\underline{r_{i}},\underline{r}\right)\;d\underline{r}=T\left(\chi \left(\underline{r}\right)\right)$$

apart from some inessential factors, where $\chi \left(\underline{r}\right)=\frac{\epsilon_{s}(\underline{r})}{\epsilon_{r_{a}}}-1$ and T are a contrast function and the pertinent linear operator for our multi-view and single-frequency scattering configurations of interest. Moreover, $G_{s}$ is the Green function pertinent to the inhomogeneous background medium and $E_{i}$ is the incident field radiated by a filamentary line source within the background medium, that is, in the presence of the dielectric cylinder. 

In particular, the Green function can be computed in closed form as a series [39] as

(2) $$G_{s}\left(\underline{r},\underline{r_{s}}\right)=\sum_{n}d_{n}^{\prime }H_{n}^{\left(2\right)}\left(\beta r_{s}\right)\;J_{n}\left(\beta \sqrt{\epsilon_{r_{a}}}\;\rho \right)\;e^{jn\left(\theta_{s}-\phi \right)}\;$$

where $H_{n}^{\left(2\right)}(\cdot )$ is the Hankel function of the second kind and *n*-th order and $J_{n}(\cdot )$ is the Bessel function of the first kind and *n*-th order. In addition, the wavenumber and wavelength are denoted by β and λ, respectively. The generalized transmission $d_{n}^{\prime }$ coefficients are provided by

(3) $$d_{n}^{\prime }=\frac{\frac{2j}{\pi \beta r_{a}}}{J_{n}\left(\beta \sqrt{\epsilon_{r_{a}}}\;r_{a}\right)\;{H_{n}^{\left(2\right)}}^{\prime }\left(\beta r_{a}\right)-\sqrt{\epsilon_{r_{a}}}\;J_{n}^{\prime }\left(\beta \sqrt{\epsilon_{r_{a}}}\;r_{a}\right)\;H_{n}\left(\beta r_{a}\right)}$$

where ${H_{n}^{\left(2\right)}}^{\prime }$ and $J_{n}^{\prime }$ are the derivative of the Hankel function and the derivative of the Bessel function, respectively. 

Because of the reciprocity theorem, the incident field $E_{i}$ in (1) is equal to the Green function $G_{s}$ (apart from an inessential constant factor), i.e., ${G_{s}\left(\underline{r},\underline{r_{i}}\right)=E}_{i}\left(\underline{r_{i}},\underline{r}\;\right)$ (see Appendix A for more details).

Note that the series in (2) can apparently be approximated using a finite summation of 2N+1 terms, where N is equal to $\left[\beta \sqrt{\epsilon_{r_{a}}}\;max(\rho )\right]$, with $\left[\cdot \right]$ representing the nearest integer. This approximation arises due to the asymptotic behavior of the Bessel function for order larger than the argument. However, the issue of the truncation of (2) will be further considered in Section 3 and in Appendix B.

### 2.1. PSF Evaluation

In this subsection, we first recall the definition of the exact PSF as the impulse response of an imaging system to a point-like scatterer and express it as the cascade of $T^{-1}$, i.e., the regularized inverse operator of T and the forward operator. In other words, the response of the system to a Dirac delta function δ is the PSF of the system. Mathematically, the exact PSF is provided by

(4) $$PSF\left(\underline{r},\underline{r_{0}}\right)=T^{-1}T\delta \left(\underline{r}-\underline{r_{0}}\right)$$

When it is observed at $\left(\underline{r}\right)$ and the point-like scatterer is located at $\left({\underline{r}}_{0}\right)$, SVD is applied to (2) because the T operator is linear and compact. Its singular system consists of the triple $\left\{v_{n},\;\sigma_{n},\;u_{n}\right\}$ [28], where $u_{n}$ and $v_{n}$ are the singular functions, which span the data and the scatterer contrast function spaces, respectively, and $\sigma_{n}$ is the singular values, arranged under a decreasing order. We can rewrite (4) in terms of the completeness relation truncated to the retained singular function $v_{n}$. This is because the minimum–norm solution to the inverse scattering problem is a projection of the actual contrast function onto the singular function having non-zero singular values.

(5) $$PSF\left(\underline{r},\underline{r_{0}}\right)=\sum_{n=1}^{N_{t}}v_{n}\left(\underline{r}\right)\;v_{n}^{*}\left(\underline{r_{0}}\right)\;$$

where ∗ indicates the conjugation operation. Equation (5) states that the exact PSF is dependent on the number of retained singular values, which is related to the accuracy of the solution. Hence, knowledge of the singular functions and the choice of $N_{t}$ are required to compute (5), and it can only be calculated in closed form for a limited number of scatterer geometries. The truncation value $N_{t}$ can be chosen in terms of the NDF, whenever the singular values exhibit a rather flat behavior before the exponentially fast decay. Then, $N_{t}$ is chosen in correspondence with the knee of the singular value curve, and it is rather independent of the noise on the data. If this is not the case, the correct choice of $N_{t}$ can be performed once the uncertainties on the data are available, as the choice of $N_{t}$ can vary and it depends on those uncertainties. Additionally, the inversion results depend on the knowledge available a priori about uncertainties on the data. Therefore, it is worth investigating the behavior of the SVD of (1) to understand what is the typical behavior of singular values for the present inhomogeneous medium geometry. 

To numerically calculate the SVD of the pertinent operator, a sufficiently fine discretization of the integral Equation (1) is employed and the resulting matrix equation is processed in the MATLAB environment. 

**Figure 2:**
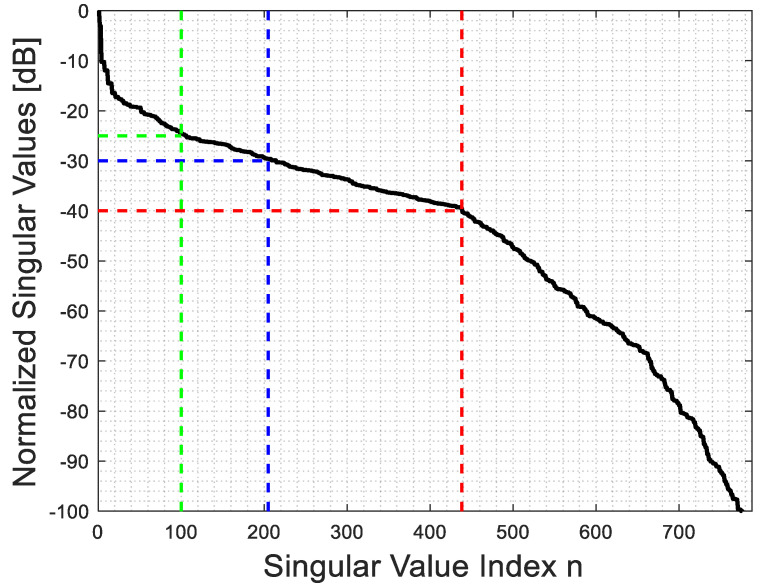
The behavior of the singular values for $\epsilon_{r_{a}}=4$ and $r_{a}=2\lambda$ for the far field.

**Figure 3:**
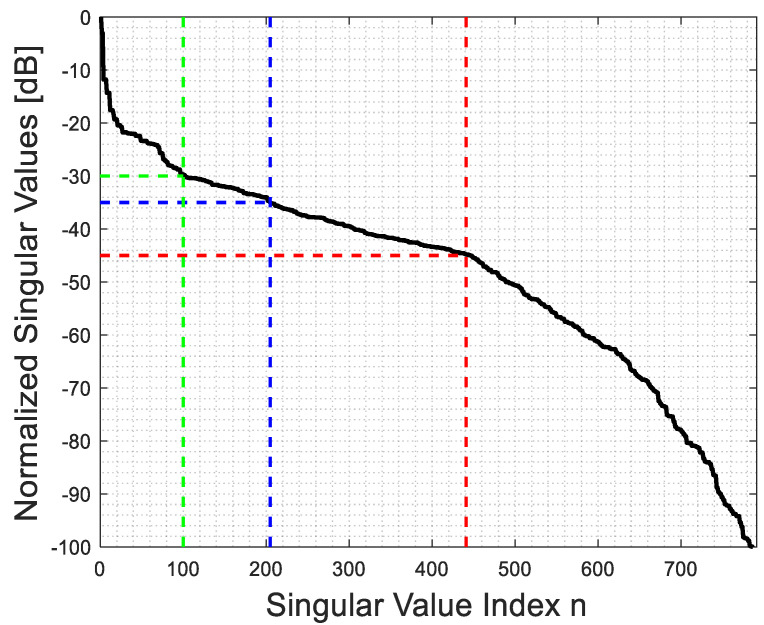
The behavior of the singular values for $\epsilon_{r_{a}}=4$ and $r_{a}=2\lambda$ for the near field.

Figure 2 and Figure 3 show the behavior of the singular values for $\epsilon_{r_{a}}=4$ and $r_{a}=2\lambda$ in the far and near fields, respectively. It is apparent that their behavior is not very different. However, in contrast to the homogeneous background medium case, where a step-like behavior can be expected due to the possibility to recast (1) as a Fourier transform [29,31], the singular value behavior now depends on $\epsilon_{r_{a}}$. Hence, it is worthwhile to examine how the truncation level of the singular values, which determines the value of $N_{t}$ in (5), impacts the behavior of PSF. 

**Figure 4:**
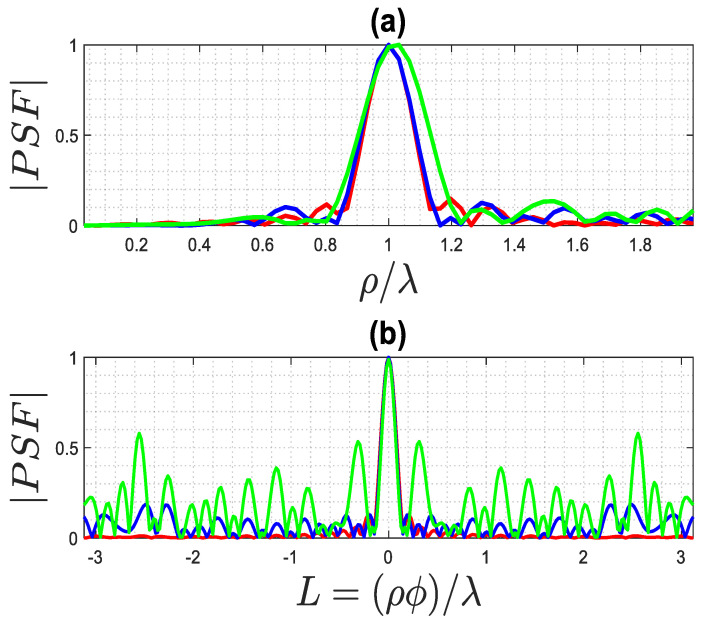
The exact PSF for far field achieved using different truncation levels, where the green line is 25 dB, the blue line is 30 dB, and the red line is 40 dB: (**a**) PSF along ρ-cut, (**b**) PSF along ϕ-cut.

**Figure 5:**
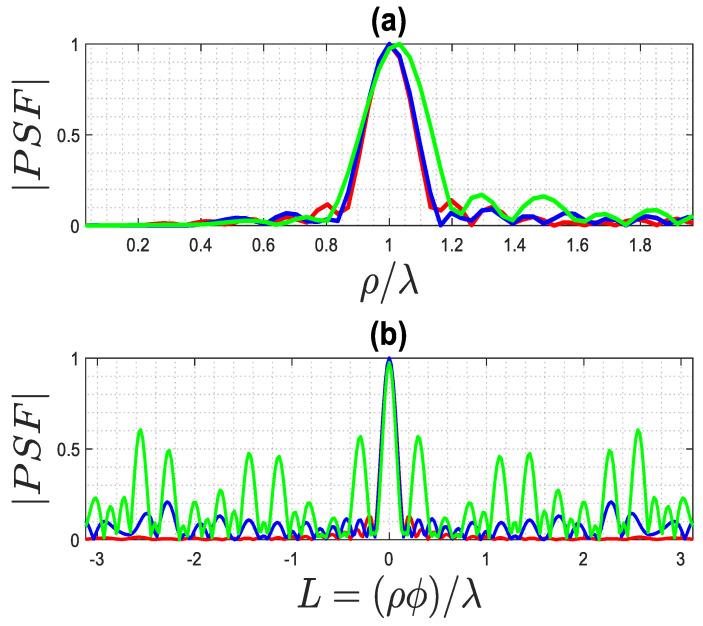
The exact PSF for the near field for different truncation levels, where the green line is 30 dB, the blue line is 35 dB, and the red line is 45 dB: (**a**) PSF along ρ-cut, (**b**) PSF along ϕ-cut.

By observing Figure 4 and Figure 5, which pertain to far and near fields (with $r_{i}=r_{s}=r_{a}+\lambda$), respectively, it can be appreciated that a high truncation level does not affect the main lobe of the PSF, while a low level considerably reduces its side lobe level. Consequently, when the main lobe of the PSF is the primary focus, as it defines the resolution of the inversion algorithm, a rather high truncation level of the singular values can be tolerated. On the contrary, when reconstructing a more complex object, such as a collection of closely located point-like scatterers, it is important to employ a low truncation level (and consequently, a low uncertainty level on data) to prevent adverse effects from high side lobes on the resulting image. 

In this paper, the achievable resolution is estimated based on the general behavior of such functions, specifically the main lobe. The resolution R is defined as half of the width W of the main lobe of the PSF function.

### 2.2. Approximate PSF 

According to [30,31,33], the adjoint operator may approximate the inverse operator in (4) if the singular values of the relevant operator have a nearly constant behavior before the knee of its curve. Consequently, it is possible to replace the inverse operator with the adjoint one in (4) to introduce a good approximation of the exact PSF to overcome the abovementioned limitation. Notwithstanding, this is not true for the case at hand; hereafter, we adopt the same approximation since it provides a simple analytical function to define the resolution.

Then, the approximated $\tilde{PSF}$ is defined by

(6) $$\tilde{PSF}\left(\underline{r},\underline{r_{0}}\right)=T^{+}T\delta \left(\underline{r}-\underline{r_{0}}\right)$$

The analytical evaluation of (6) is performed as follows. First, we define the adjoint operator of (1) by

(7) $$T^{+}E_{s}=\int_{0}^{2\pi }\int_{0}^{2\pi }E_{s}(\underline{r_{s}},\underline{r_{i}})\;G_{s}^{*}\left(\underline{r_{0}},\underline{r_{s}}\right)E_{i}^{*}\left(\underline{r_{i}},\underline{r_{0}}\right)\;d\theta_{s}\;d\theta_{i}$$

as the source and observation domains are assumed to be the circumference. Then, the spectral theorem for compact self-adjoint operators is applied to $T^{+}T$:

(8) $$T^{+}T\delta \left(\underline{r}-\underline{r_{0}}\right)=\;\iint_{ID}^{}\chi (\underline{r})\left[\int_{0}^{2\pi }\int_{0}^{2\pi }G_{s}\left(\underline{r},\underline{r_{s}}\right)\;E_{i}\left(\underline{r_{i}},\underline{r}\right)\;G_{s}^{*}\left(\underline{r_{0}},\underline{r_{s}}\right)E_{i}^{*}\left(\underline{r_{i}},\underline{r_{0}}\right)\;d\theta_{s}\;d\theta_{i}\;\right]\;d\underline{r}$$

By using the addition theorem for the Hankel function and interchanging the integrals and the summations, because of (2), (8) becomes a four-fold summation, which, in turn, can be factored as the product of two identical functions with different arguments:

(9) $$\tilde{PSF}\left(\rho ,\rho_{0},\phi ,\phi_{0}\right)=F_{s}\left(\rho ,\rho_{0},\phi ,\phi_{0},r_{s}\right)\cdot F_{i}\left(\rho ,\rho_{0},\phi ,\phi_{0},r_{i}\right)$$

where $F_{s}\left(\rho ,\rho_{0},\phi ,\phi_{0},r_{s}\right)$ and $F_{i}\left(\rho ,\rho_{0},\phi ,\phi_{0},r_{i}\right)$ pertain to the observation and incident field, respectively, and are symmetric functions of the arguments. For the full-view case, for instance, the $F_{s}$ function can be written as a double summation:

(10) $$F_{s}\left(\rho ,\rho_{0},\phi ,\phi_{0},\;r_{s}\right)=\int_{0}^{2\pi }G_{s}\left(\underline{r},\underline{r_{s}}\right)G_{s}^{*}\left(\underline{r_{0}},\underline{r_{s}}\right)d\theta_{s}=\;\left(\begin{array}{c} \int_{0}^{2\pi }\;\sum_{n=-N}^{N}d_{n}^{\prime }\;H_{n}^{\left(2\right)}\left(\beta r_{s}\right)\;J_{n}\left(\beta \sqrt{\epsilon_{r_{a}}}\;\rho \right)\;e^{jn\left(\theta_{s}-\phi \right)}\;.\\ \;{\left(\sum_{l=-L}^{L}d_{l}^{\prime }\;H_{l}^{\left(2\right)}\left(\beta r_{s}\right)\;J_{l}\left(\beta \sqrt{\epsilon_{r_{a}}}\;\rho_{0}\right)\;e^{jl\left(\theta_{s}-\phi_{0}\right)}\right)}^{*}d\theta_{s} \end{array}\right)$$

Then, since the observation domain is a circumference, so that $r_{s}$ is constant, by performing the simple closed form integration, (10) becomes

(11) $$F_{s}\left(\rho ,\rho_{0},\phi ,\phi_{0},r_{s}\right)=\sum_{n=-N}^{N}{\left|d_{n}^{\prime }\right|}^{2}\;{\left|H_{n}^{\left(2\right)}\left(\beta r_{s}\right)\right|}^{2}J_{n}\left(\beta \sqrt{\epsilon_{r_{a}}}\;\rho \right)\;J_{n}\left(\beta \sqrt{\epsilon_{r_{a}}}\;\rho_{0}\right)\;e^{jn\left(\phi -\phi_{0}\right)}$$

The evaluation of $F_{i}$ proceeds in the same way as $F_{s}$. Finally, the evaluation of (9) is given by

(12) $$\tilde{PSF}\left(\rho ,\rho_{0},\phi ,\phi_{0}\right)=\left[\sum_{n=-N}^{N}{\left|d_{n}^{\prime }\right|}^{2}\;{\left|H_{n}^{\left(2\right)}\left(\beta r_{s}\right)\right|}^{2}J_{n}\left(\beta \sqrt{\epsilon_{r_{a}}}\;\rho \right)\;J_{n}\left(\beta \sqrt{\epsilon_{r_{a}}}\;\rho_{0}\right)\;e^{jn\left(\phi -\phi_{0}\right)}\right].\;\left[\sum_{m=-M}^{M}{\left|d_{m}^{\prime }\right|}^{2}\;J_{m}\left(\beta \sqrt{\epsilon_{r_{a}}}\;\rho \right)\;{\left|H_{m}^{\left(2\right)}\left(\beta r_{i}\right)\right|}^{2}J_{m}\left(\beta \sqrt{\epsilon_{r_{a}}}\;\rho_{0}\right)\;e^{jm\left(\phi -\phi_{0}\right)}\right]$$

which provides the searched analytical evaluation of the approximated $\tilde{PSF}$. Although (12) provides a closed-form expression under a finite series, further simplifications are considered hereafter.

## 3. Discussion about the Approximated $\tilde{PSF}$

In this section, we provide a further discussion about the approximated $\tilde{PSF}$ (12) to simplify it and to demonstrate that the resolution is the same for both the near and far scattered fields. In particular, we assume that the ID coincides with the whole circular section or region 1 in Figure 1, so that $\max\left(\rho ,\rho_{0}\right)=r_{a}$. Therefore, we consider the influence of ${\left|H_{n}^{\left(2\right)}\left(\beta r_{s}\right)\right|}^{2}$, ${\left|d_{n}^{\prime }\right|}^{2}$ and $c_{n}={\left|J_{n}(\beta \sqrt{\epsilon_{r_{a}}}\;r_{a})\right|}^{2}$ in (12) on the resolution. 

### 3.1. Far Field

**Figure 6:**
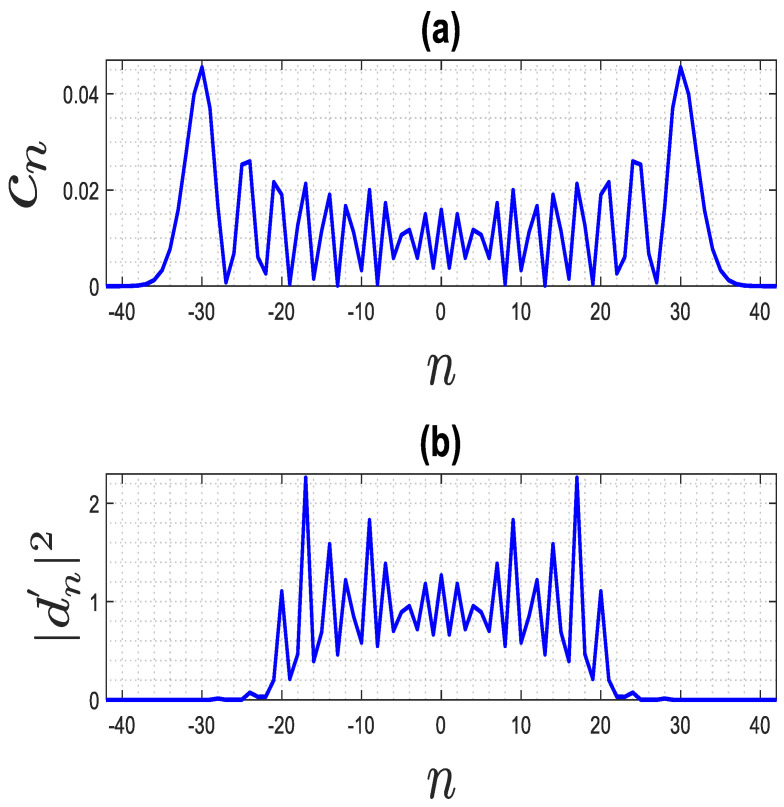
The behavior of two coefficients in (12): (**a**) the behavior of $c_{n}$, (**b**) the behavior of ${\left|d_{n}^{\prime }\right|}^{2}$.

For the far field, due to the asymptotic behavior of the Hankel functions for arguments much larger than the order, ${\left|H_{n}^{\left(2\right)}\left(\beta r_{s}\right)\right|}^{2}$ can be approximated by $\frac{\pi }{2(\beta r_{s})}$, which becomes a constant. Next, the influence of ${\left|d_{n}^{\prime }\right|}^{2}$ on the behavior of the Fourier coefficients in (12) needs to be examined. To this end, Figure 6b shows a typical behavior of ${\left|d_{n}^{\prime }\right|}^{2}$ for $r_{a}=3\lambda$ $\epsilon_{r_{a}}=3$. It can be observed that they decay for large n (for an explanation, see Appendix B) and that their amplitude is mostly close to 1. On the other hand, as discussed in Section 2 and confirmed by Figure 6a, the $c_{n}$ coefficients decay asymptotically for n>N. Therefore, it is interesting to examine the behavior of the product $\left|d_{n}^{\prime }\;J_{n}\left(\beta \sqrt{\epsilon_{r_{a}}}\;r_{a}\right)\right|$ (as $\max\left(\rho ,\rho_{0}\right)=r_{a}$). In the Appendix B, it is shown that this term decays asymptotically for $\left|n\right|>N^{\prime }=\left[\beta \;r_{a}\right]$. Consequently, the Fourier series in (12) can be truncated to ${2N}^{\prime }+1$ terms as

(13) $$\tilde{PSF}\left(\rho ,\rho_{0},\phi ,\phi_{0}\right)≅{\left(\sum_{n=-N^{\prime }}^{N^{\prime }}J_{n}\left(\beta \sqrt{\epsilon_{r_{a}}}\;\rho \right)\;J_{n}\left(\beta \sqrt{\epsilon_{r_{a}}}\;\rho_{0}\right)\;e^{jn\left(\phi -\phi_{0}\right)}\right)}^{2}\;$$

But, in virtue of the addition theorem of the Bessel functions, (13) is approximately equal to

(14) $$\tilde{PSF}\left(\rho ,\rho_{0},\phi ,\phi_{0}\right)≅\;{\left(J_{0}\left(\beta \sqrt{\epsilon_{r_{a}}}\;\left|\underline{r}-\underline{r_{0}}\right|\right)\right)}^{2}\;$$

**Figure 7:**
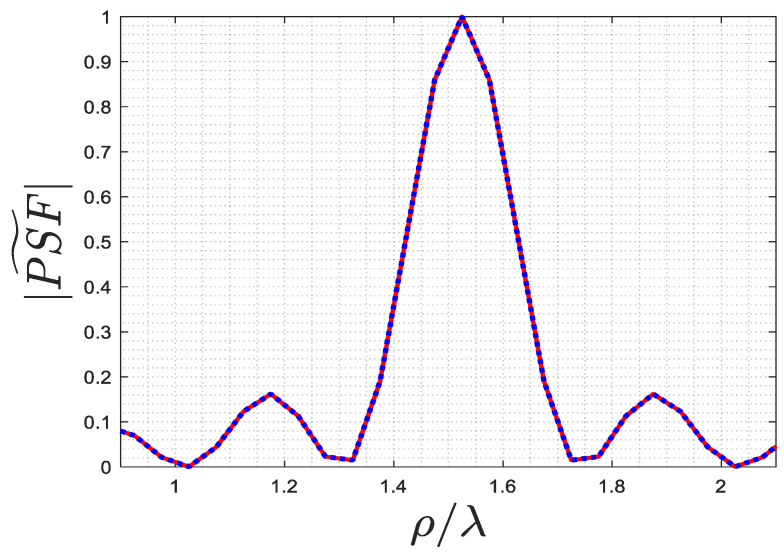
The comparison of the normalized amplitude of (12) (solid blue line) and (14) (dotted red line) PSFs along ρ-cut for $\rho_{0}=1.525\lambda$ and $\phi_{0}=0$.

A comparison between (12) and (14) is provided in Figure 7 for $\rho_{0}=1.52\lambda$ and $\phi_{0}=0$. The results confirm that the two approximations are completely overlapped.

### 3.2. Near Field

**Figure 8:**
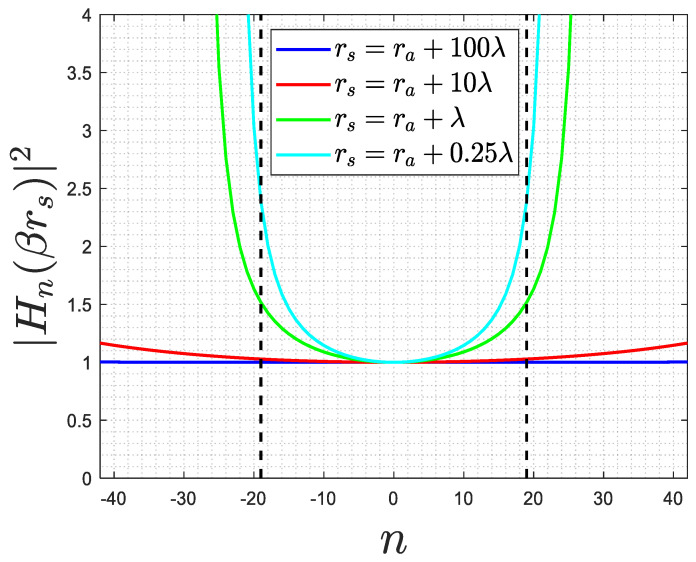
The behavior of ${\left|H_{n}^{\left(2\right)}\left(\beta r_{s}\right)\right|}^{2}$ for different $r_{s}$ (dashed lines shows the value of *N’*).

The results obtained for ${\left|d_{n}^{\prime }\right|}^{2}$ are also valid for the near field as they are independent of $r_{s}$. Therefore, the influence of the ${\left|H_{n}^{\left(2\right)}\left(\beta r_{s}\right)\right|}^{2}$ factor needs to be considered. Figure 8 shows a comparison of ${\left|H_{n}^{\left(2\right)}\left(\beta r_{s}\right)\right|}^{2}$ for different $r_{s}$. It is observed that while the curve remains flat for large $r_{s}$ values, this is not the case for smaller values. However, even when $r_{s}=a+\lambda$, it can still be considered flat for $\left|n\right|<N\prime$, thereby having a negligible impact on (12), and (13) and (14) still hold. Consequently, it can be concluded that the approximate PSF is the same for both the near and far fields, except for cases where $r_{s}<r_{a}+0.5\lambda$, i.e., very close to the dielectric ID. In such cases, it becomes necessary to consider more terms for the convergence of the Fourier series and to account for the close proximity effects of the reactive near field, which become significant.

**Figure 9:**
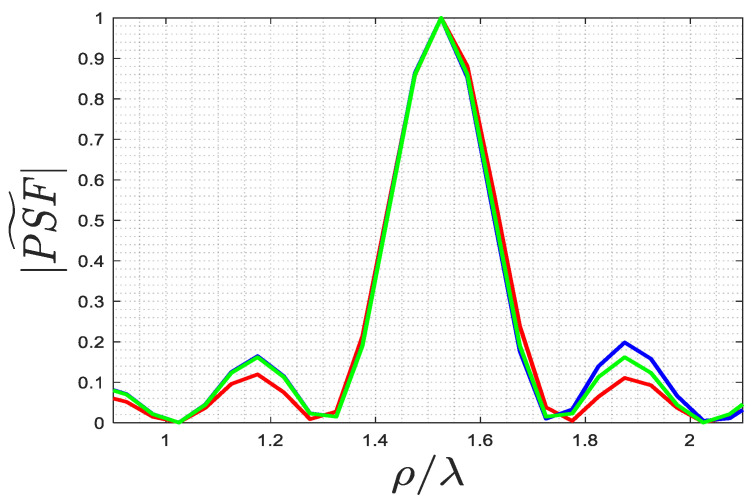
The comparison of the normalized amplitude of (12) (red line), (13) (blue line), and (14) (green line) PSFs along ρ-cut for $\rho_{0}=1.525\lambda$ when $\phi_{0}=0$.

Figure 9 shows a comparison of Equations (12)–(14) for $\rho_{0}=1.52\lambda$ and $\phi_{0}=0$. The results verify that the three approximations coincide with each other, as expected. Based on the results obtained from the two subsections, it can be concluded that (14) can serve as a reliable approximation for the exact PSF instead of (12). Additionally, it is notable that the resolution remains the same for both the far and near fields. Further numerical examples will be provided in the next section.

## 4. Numerical Validation

In this section, various numerical examples are presented to validate the theoretical discussions from the previous sections. We consider a cylinder with a radius of $r_{a}=3\lambda$, where the ID coincides with the cylinder. To highlight the focusing properties, only the main lobe of the PSF is taken into account, and the amplitudes of both PSFs are normalized to 1. For all subsequent numerical examples, $r_{i}$ and $r_{s}$ are set to $r_{a}+\lambda$ for the near field.

**Figure 10:**
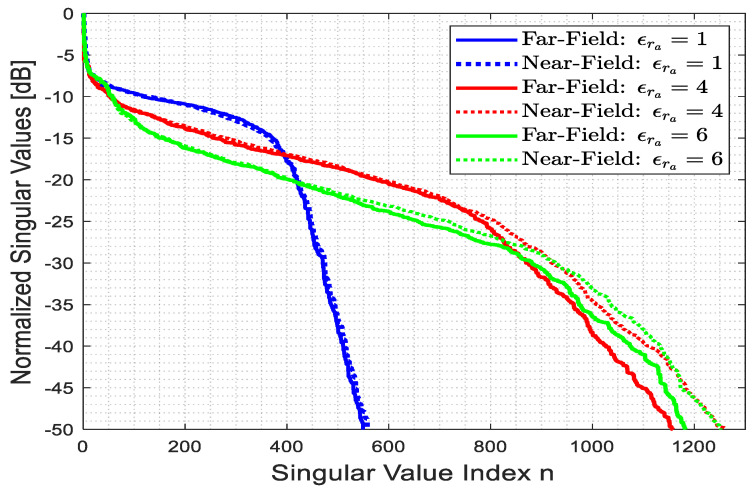
The behavior of the normalized singular values of the linearized inverse scattering for different $\epsilon_{r_{a}}$ for the far and near fields.

Firstly, we compare the behavior of singular values of (1) for the far and near fields with different $\epsilon_{r_{a}}$. Figure 10 illustrates the behavior of normalized singular values of the relevant operators (1) for the near and far fields with varying $\epsilon_{r_{a}}$. The analytical estimation of the NDF for a free space was provided in [40,41] for the far field, and it is provided using $NDF=\frac{\Sigma A}{{\left(2\pi \right)}^{2}}$, where A and Σ are the spectral domain area and the measure of the area of the function to be transformed, respectively. For the full-view case, A is $\pi {\left(2\beta \right)}^{2}$ [41] and Σ is equal to $\pi {r_{a}}^{2}$ for the considered ID, and the NDF estimation is confirmed via the blue solid line. The results provide evidence that the singular value behavior is approximately the same for the far and near fields. In addition, the singular value behavior is not flat, indicating that higher values of $\epsilon_{r_{a}}$ result in a faster overall decay.

**Figure 11:**
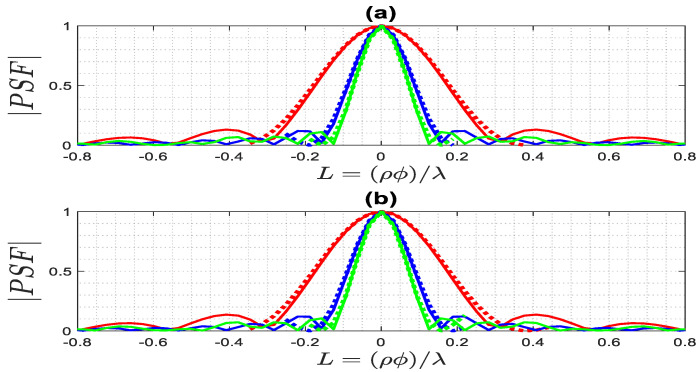
The comparison of the normalized amplitude of the exact (solid lines) and approximated (dashed lines) PSFs along a ϕ-cut for $\phi_{0}=0$ and $\rho_{0}=1.5\lambda$, for $\epsilon_{r_{a}}=1$ (red lines), $\epsilon_{r_{a}}=4$ (blue lines) $\epsilon_{r_{a}}=6$ (green lines): (**a**) far field, (**b**) near field.

A comparison between the exact PSF (12) and the approximated (14) one is performed for both the far and near fields to further evaluate the performance of the achievable resolution and to validate the accuracy of the approximated $\tilde{PSF}$. Figure 11 illustrates the normalized amplitude of both PSFs along the ϕ-cuts when $\rho_{0}=1.5\lambda$ and $\phi_{0}=0$. It is observed that the resolution is the same for both the far and near fields for different $\epsilon_{r_{a}}$, as the main lobe width of the PSF becomes slimmer as $\epsilon_{r_{a}}$ increases. Therefore, the resolution R for $\epsilon_{r_{a}}=1$, $\epsilon_{r_{a}}=4$, and $\epsilon_{r_{a}}=6$ is equal to 0.38λ, 0.19λ, and 0.15λ, respectively, as it can be predicted using (14) according to the first zero of the Bessel function of *0*-th order. The space-invariance of the PSF being achieved for the full-view case means that the resolution is constant. This result confirms that two PSFs are approximately overlapped.

**Figure 12:**
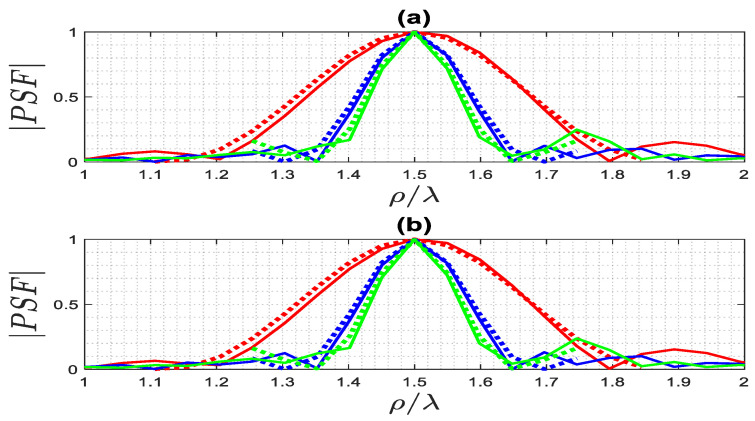
The comparison of the normalized amplitude of the exact (solid lines) and approximated (dashed lines) PSFs along a ρ-cut for $\rho_{0}=1.5\lambda$ when $\phi_{0}=0$, for $\epsilon_{r_{a}}=1$ (red lines), $\epsilon_{r_{a}}=4$ (blue lines) $\epsilon_{r_{a}}=6$ (green lines): (**a**) far field, (**b**) near field.

To check out the performance of the exact PSF (12) and the approximated (14) along a ρ-cut for the far and near fields, a comparison between two PSFs is provided in Figure 12 for $\rho_{0}=1.5\lambda$ when $\phi_{0}=0$. The resolution is again the same for both the far and the near fields for different $\epsilon_{r_{a}}$, as expected. In addition, it is confirmed that the approximated $\tilde{PSF}\;$ works well. As a result, the resolution is proportion to $\frac{1}{\sqrt{\epsilon_{r_{a}}}}$.

## 5. Application to Breast Cancer Scenario

This section provides an application of the aforementioned theoretical discussions to reconstruct a set of point-like scatterers located within an ID (blue circle) with $r_{a}=1.5\lambda$ and $\epsilon_{r_{a}}=12$ from the near-field scattered data ($r_{i}=r_{s}=a+\lambda$). We are aware that actual breast cancer scenarios are more complicate, with strongly inhomogeneous background media, which prevent any analytical work and require numerical modelling. However, in this paper, the goal is to provide an analytical discussion of the resolution and some simplifications are required, such as assuming a dielectric homogenous cylindrical circular investigation domain and modeling tumors as point-like scatterers. For this case, the truncation level of the singular values is selected at 40 dB. As discussed in Section 3, this choice ensures a low side lobe level for the exact PSF without affecting its main lobe. The ID is chosen to mimic a breast cancer scenario, where the dielectric background consists of a medium with high dielectric permittivity, approximating a circular shape. Breast imaging aims to identify the presence of breast cancer or tumors, and it has been extensively studied in the field of microwave sensing and imaging. In particular, breast tumors exhibit relatively high contrast compared with the predominating fat tissue in the breast [42] and can be modelled as point-like scatterers. Therefore, this approach can be used for localizing or detecting breast cancer/tumors, as accurate detection is the first step in classifying cancer/tumors.

**Figure 13:**
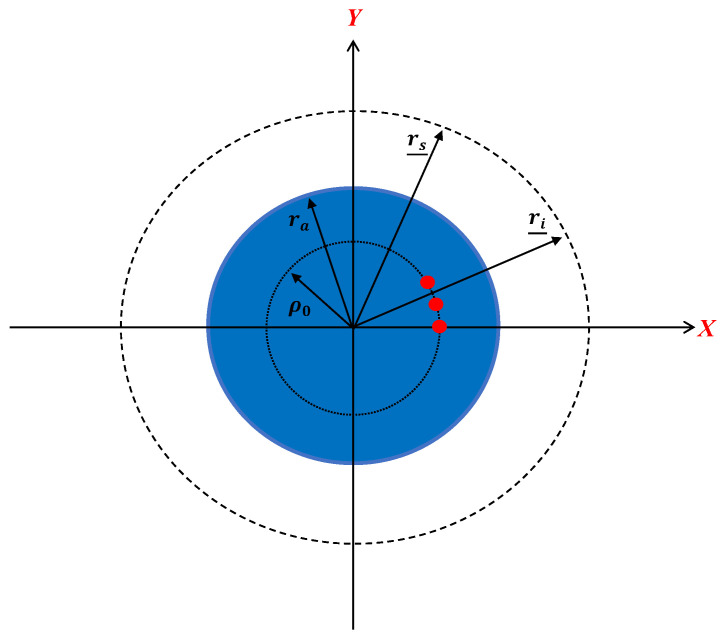
The geometry of the application. The red dots indicate the position of the point-like scatterers. The dotted line indicates the positions of source and receivers.

In this application, we consider three point-like scatterers on a circumference (the ϕ-cut) with a radius of $\rho_{0}=0.26\lambda$ located in the ID, representing a typical size of the array for breast imaging. Figure 13 shows the geometry of the application.

**Figure 14:**
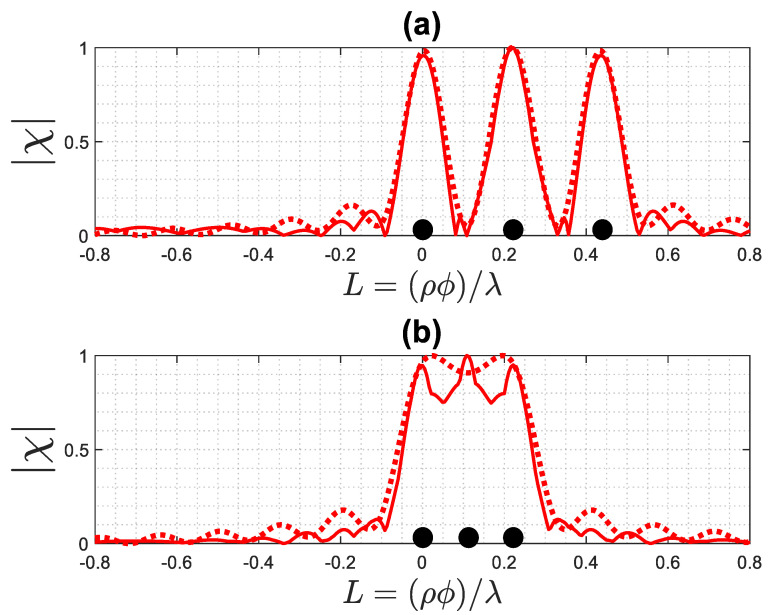
The exact (solid line) and approximated (dotted line) reconstruction of three point-like scatterers: (**a**) the distance between them is equal to the resolution, (**b**) the distance between them is less than the resolution. The black dots indicate the position of the point-like scatterers.

Figure 14 shows a normalized reconstruction of the considered point-like scatterers, computed via inversion of (1), compared with the result of the summation of three functions (14) as centered at the scatterers positions. As can be seen in Figure 14a, if the distance between point-like scatterers is equal to the width W=0.2λ, they can be distinguished from each other, as the predicted resolution is R=0.1λ. However, when the distance is less than *R*, they are not resolvable and appear as a single scattering point, as shown in Figure 14b. The findings indicate that both reconstructions yield similar results.

**Figure 15:**
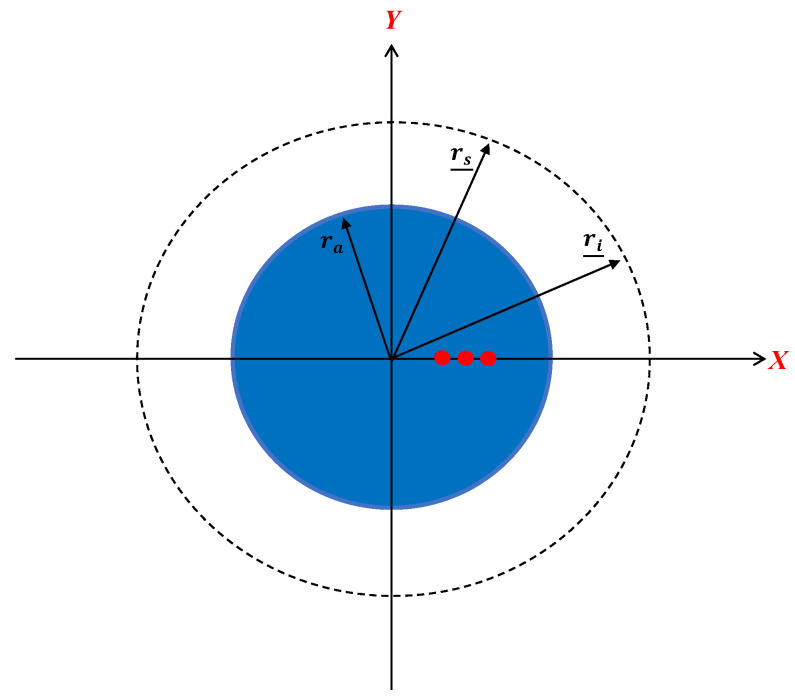
The geometry of the application. The red dots indicate the position of the point-like scatterers. The dotted line indicates the positions of source and receivers.

Now, we consider an example where the three point-like scatterers are arranged along the radius of the ID when $\phi_{0}=0$, as shown in Figure 15.

**Figure 16:**
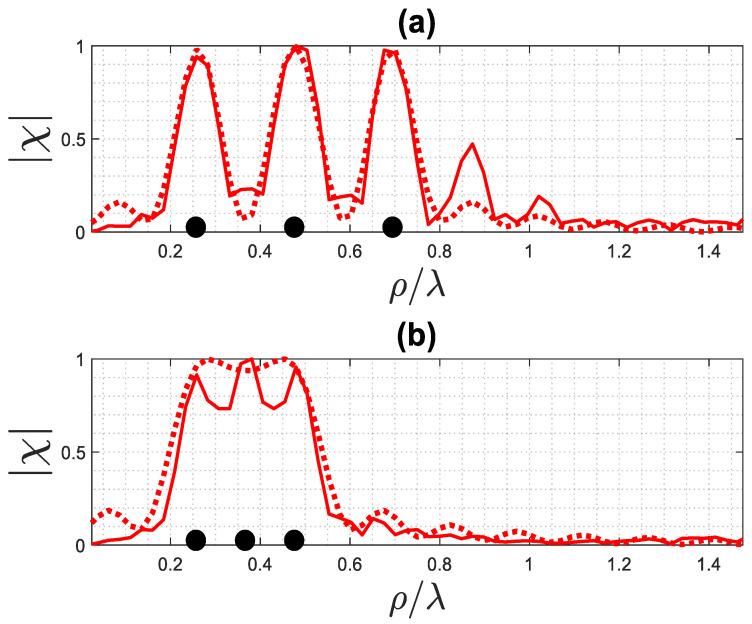
The exact (solid line) and approximated (dotted line) reconstruction of three point-like scatterers: (**a**) the distance between them is equal to the width, and (**b**) the distance between them is equal to the resolution. The black dots indicate the position of the point-like scatterers.

Figure 16 displays the normalized reconstruction of the point-like scatterers under examination computed via inversion of (1), compared with the result of the summation of three functions (14). Figure 16a demonstrates that, if the distance between the scatterers is the same as the width W=0.2λ, they can be identified separately. On the other hand, if the distance is smaller than the resolution, with R=0.1λ, they are not distinguishable and appear as a single scattering point, as depicted in Figure 16b. These results once again highlight that both reconstructions produce comparable outcomes.

## 6. Discussion and Conclusions

We have evaluated the PSF of the linear inverse scattering problem for a dielectric cylinder background to estimate the achievable resolution for the full-view case for both the far and near fields. Our main goal has been to provide an analytical approximation of the resolution because the exact evaluation of the PSF can be accomplished only numerically and its accuracy is dependent on the truncation value. First, we have discussed the behavior of the singular values and showed that the singular value behavior is approximately the same for both fields. However, since their behavior is not flat, the accuracy of the result can depend on the number of singular values to be retained, which, in turn, depends on the uncertainties on data. It has been pointed out that the choice of the truncation level for the PSF computation affects only its side lobe level, while the main lobe remains mostly unchanged. Then, an approximate analytical PSF has been introduced and some numerical simulations have validated the accuracy of the approximated PSF against the exact PSF. In particular, the results have shown that there is good agreement in the main lobe region of both PSFs for different permittivities, which is sufficient for predicting actual resolution in a sensing configuration. The analytical and the numerical results have also demonstrated that the resolution is the same for both fields and remains unchanged along the whole ID, i.e., it is space-invariant. Additionally, both results clearly have highlighted that the resolution changes by varying the permittivity of the ID in both fields and is inversely proportional to $\sqrt{\epsilon_{r_{a}}}$.

Finally, we have presented an application for reconstructing point-like scatterers located within the ID from the near field, which is valuable for detecting breast cancer/tumors. The application demonstrates that when the distance between two point-like scatterers equals the width of the main lobe PSF, they can be distinguished from each other. Conversely, if the distance is less than the width, they cannot be differentiated. The results once again have shown that both reconstructions achieved similar results. Indeed, the approach suffers from a limitation. In fact, the approximate evaluation of the PSF is shown to be accurate in the main lobe, while the behavior of the side lobes is less predictable. This means that the reconstruction of the isolated point-like scatterers, even if randomly located, can be expected to be accurate. On the contrary, if there are many point-like scatterers and they are close to each other, it may not be possible to reconstruct all of them correctly due to the effect of the side lobes of the corresponding approximate PSF.

For microwave imaging systems designed to operate up to 2GHz, for a reasonable value of the relative permittivity of human tissues as $\epsilon_{r_{a}}$= 6, a resolution of 2.25 cm can be predicted. Of course, from the imaging point of view, this figure can provide unsatisfactory results in actual complicated scenarios with many tumors with smaller separations. However, for detection purposes, especially in the very initial stage, the results of the presented analysis can provide the minimum detection distance for isolated tumors. In any case, the resolution at microwave frequencies is connected to the free space wavelength because of the wave scattering interaction.

## Data Availability

Data supporting the reported results were generated during the study.

## Appendix A

In this appendix, we demonstrate the connection between the Green function and the incident field by starting from equations (7-32a) in [43], which provide an integral relating the file radiated in the region 2 by sources occupying region 1 to the field radiated in region 1 by sources occupying region 2. In the scalar case, where both electric sources and fields have only the z-component, if we assume no magnetic sources and electric filamentary line sources with a constant current, the integrals can be evaluated straightforwardly.

Then, the source 1, $J_{1}=I\delta \left(\underline{r\prime }-\underline{r_{s}}\right)$, located in $\underline{r\prime }=\underline{r_{s}}$, radiates the field $E_{1}\left(\underline{r\prime }\right)=E_{i}\left(\underline{r_{s}},\underline{r\prime }\right)$, while the source 2 $,J_{2}=\delta \left(\underline{r\prime }-\underline{r}\right)$, located in $\underline{r\prime }=\underline{r}$, radiated the field $E_{2}\left(\underline{r\prime }\right)=G_{s}\left(\underline{r},\underline{r\prime }\right)$. Consequently, the left-hand side of equations (7-32a) becomes $IG_{s}\left(\underline{r},\underline{r_{s}}\right)$ and the right-hand side becomes $E_{i}\left(\underline{r_{s}},\underline{r}\right)$, and the two functions are proportional when the argument variables are exchanged.

## Appendix B

In this appendix, we examine the asymptotic behavior for large *n* of the Fourier coefficients of (12) in the far-field case. In particular, we consider the factor $\left|d_{n}^{\prime }\;J_{n}\left(\beta \sqrt{\epsilon_{r_{a}}}\;\rho \right)\right|$, which is common to all summations. We make use of the following asymptotic expansion of the Bessel and Hankel functions for orders larger than the arguments:

(A1) $$J_{n}\left(z\right)\sim \frac{1}{\sqrt{2\pi n}}{\left(\frac{ez}{2n}\right)}^{n}$$

and

(A2) $$H_{n}^{\left(2\right)}\left(z\right)\sim j\sqrt{\frac{2}{\pi n}}{\left(\frac{ez}{2n}\right)}^{-n}$$

where e is Euler’s number.

We start from the denominator of (3), to be rewritten as

(A3) $$J_{n}\left(\beta \sqrt{\epsilon_{r_{a}}}\;r_{a}\right)H_{n-1}^{\left(2\right)}\;\left(\beta r_{a}\right)-\sqrt{\epsilon_{r_{a}}}\;J_{n-1}\left(\beta \sqrt{\epsilon_{r_{a}}}\;r_{a}\right)\;H_{n}\left(\beta r_{a}\right)$$

in virtue of the Bessel functions. Then, via substitution of (A1) and (A2), we obtain

(A4) $$\frac{\sqrt[{n}]{\epsilon_{r_{a}}}}{\pi }\frac{2}{e\beta r_{a}}\frac{n^{n}}{{\left(n-1\right)}^{n-1}}\left\{{\left[\frac{e\beta r_{a}}{2}{\left(\frac{n-1}{n}\right)}^{n}\frac{1}{n-1}\right]}^{2}-1\right\}$$

which can be simplified to

(A5) $$\frac{\sqrt[{n}]{\epsilon_{r_{a}}}}{\pi }\frac{2}{e\beta r_{a}}$$

This provides

(A6) $$\left|d_{n}^{\prime }\right|\sim \frac{e}{\sqrt[{n}]{\epsilon_{r_{a}}}}$$

When we multiply this factor by the asymptotic expansion of the Bessel function, we obtain

(A7) $$\left|d_{n}^{\prime }J_{n}\left(\beta \sqrt{\epsilon_{r_{a}}}\;\rho \right)\right|\sim \frac{e}{\sqrt{2\pi n}}{\left(\frac{e\beta \rho }{2n}\right)}^{n}\sim e\left|J_{n}\left(\beta \;\rho \right)\right|$$

This means that the product decays asymptotically for $\left|n\right|>\max\left(\beta \;\rho \right)=\left[\beta r_{a}\right]=N^{\prime }.$
